# Sequencing of cerebrospinal fluid cell-free DNA facilitated early differential diagnosis of intramedullary spinal cord tumors

**DOI:** 10.1038/s41698-024-00541-w

**Published:** 2024-02-22

**Authors:** Ruichao Chai, Songyuan An, Han Lin, Bo Pang, Hao Yan, Yun Liu, Yilin Wu, Long Wang, Xing Liu, Huiyuan Chen, Xueyu Yang, Qing Chang, Wenqing Jia, Yongzhi Wang

**Affiliations:** 1 Department of Molecular Neuropathology, Department of Neuropathology, Beijing Neurosurgical Institute, Beijing Tiantan Hospital, Capital Medical University, Beijing, China; 2 Department of Neurosurgery, Beijing Tiantan hospital, Capital Medical University, Beijing, China; 3 State Key Laboratory of Neurology and Oncology Drug Development, Jiangsu Simcere Pharmaceutical Co., Ltd., Jiangsu Simcere Diagnostics Co.,Ltd., Nanjing, China

**Keywords:** CNS cancer, CNS cancer, Diagnostic markers, Molecular medicine, Oncogenesis

## Abstract

Pre-surgery differential diagnosis is valuable for personalized treatment planning in intramedullary spinal cord tumors. This study assessed the performance of sequencing cell-free DNA (cfDNA) in cerebrospinal fluid (CSF) for differential diagnosis of these tumors. Prospectively enrolling 45 patients with intramedullary spinal cord lesions, including diffuse midline glioma (DMG), H3K27-altered (14/45), glioblastoma (1/45), H3-wildtype-astrocytoma (10/45), ependymoma (11/45), and other lesions (9/45), CSF samples were collected via lumbar puncture (41/45), intraoperative extraction (3/45), and Ommaya reservoir (1/45). Then, these samples underwent targeted sequencing along with paired tissue DNA. DMG, H3K27-altered patients exhibited a higher ctDNA positivity (85.7%, 12/14) compared to patients with H3-wildtype-astrocytoma (0/8, *P* = 0.0003), ependymoma (2/10, *P* = 0.003), and glioneuronal tumor (0/3, *P* = 0.009). The histological-grade-IV (*P* = 0.0027), Ki-67 index ≥10% (*P* = 0.014), and tumor reaching spinal cord surface (*P* = 0.012) are also associated with higher ctDNA positivity. Interestingly, for patients with TERT promoter mutant tumors, TERT mutation was detectable in the CSF cfDNA of one DMG case, but not other five cases with histological-grade-II tumors. Shared copy number variants were exclusively observed in DMG, H3K27-altered, and showed a strong correlation (Correlation = 0.95) between CSF and tissue. Finally, H3K27M mutations in CSF exhibited high diagnostic efficiency for DMG, H3K27-altered (Sensitivity = 85.7%, Specificity = 100.0%, AUC = 0.929). Notably, H3K27M was detectable in CSF from patients with recurrent tumors, making it easily applicable for postoperative monitoring. In conclusion, the molecular profile from ctDNA released into CSF of malignant tumors was more frequently detected compared to relatively benign ones. Sequencing of ctDNA in CSF exhibited high efficiency for the differential diagnosis of DMG, H3K27-altered.

## Introduction

Intramedullary spinal cord tumors display considerable heterogeneity, manifesting distinct survival times depending on varied pathological classifications^1–3^. Typically, patients undergo imaging, followed by either a biopsy alone or a biopsy combined with resection for pathological diagnosis. However, due to the sensitive and highly functional nature of the spinal cord, invasive procedures such as biopsies or surgeries carry the risk of damaging nerve function, and intramedullary tumors pose a higher risk for surgery than extramedullary tumors^4,5^. Furthermore, our prior research revealed that an extensive resection significantly enhanced the survival outcomes for patients with H3-wildtype spinal cord tumors, whereas it did not confer the same benefit for those with H3K27-altered tumors.^6–8^. Genetic alterations in spinal cord tumors have also been linked to the rate of tumor-associated spinal cord injury and subsequent functional impairment^9^. In such cases, pre-surgery differential diagnosis plays a crucial role in tailoring personalized treatment plans for patients with spinal cord tumors and other non-tumor lesions.

Utilizing cell-free DNA (cfDNA), liquid biopsy shows significant promise as a method for early differential diagnosis and grading of tumors. This approach enables the detection of circulating tumor DNA (ctDNA) released by tumors, representing a crucial component of cfDNA^10–12^. For central nervous system tumors, such as medulloblastoma and diffuse intrinsic pontine glioma characterized by aggressive growth patterns, molecular profiling of cerebrospinal fluid (CSF) cfDNA has shown superior performance compared to blood plasma analysis. This superiority is primarily attributed to the presence of the blood-brain barrier^13,14^. Nevertheless, the efficacy of cfDNA detection in CSF for differentially diagnosing spinal cord tumors in a relatively large cohort remains uncertain, given their rarity^15^.

Here, we collected cerebrospinal fluid (CSF) samples prospectively from 45 patients with spinal cord lesions to assess the effectiveness of sequencing cell-free DNA (cfDNA) in CSF for pre-surgery differential diagnosis of spinal cord tumors. The CSF samples underwent targeted sequencing of 131 genes, along with paired tissue DNA. We revealed CSF from patients with malignant tumors exhibited a higher rate of positive ctDNA compared to those with more benign tumors through panel sequencing. DMG, H3K27-altered patients also had a higher frequency of shared mutations between CSF and tissues. We also found that the shared copy number variants (CNVs) between CSF and tumor tissue, including PDGFRA, MDM2, CDK4, and others, were exclusively observed in DMG, H3K27-altered, and the copy number value of shared CNVs showed a strong correlation between CSF and tissue. Moreover, the presence of H3K27M mutations in CSF demonstrated a high diagnostic efficiency in predicting DMG, H3K27-altered. Notably, the H3K27M mutation could be detected in CSF of recurrent H3K27M-mutant tumors. Overall, sequencing cfDNA in CSF demonstrated promising results for the differential diagnosis of spinal cord DMG, H3K27-altered.

## Results

### Clinical and pathological characteristics of enrolled patients

**Fig. 1:**
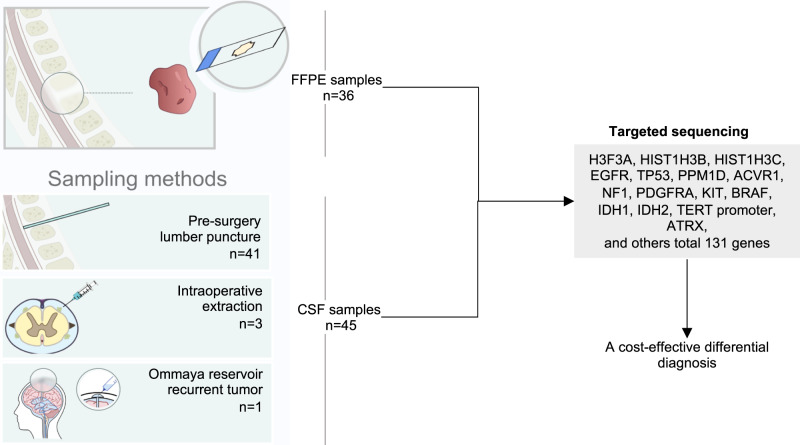
Design and workflow of this study. A total of 45 CSF samples were collected, including 39 samples from patients with tumors and 6 samples from patients with non-tumor lesions. In addition, 36 paired FFPE tumor samples were also collected. The cell-free DNA in CSF and genomic DNA from tumor tissues were utilized to perform targeted-sequencing on 131 genes. The full gene list in the Supplementary Table 1.

Totally, we obtained the cerebrospinal fluid (CSF) samples from 45 spinal cord patients via preoperative lumbar puncture (41/45), intraoperative extraction (3/45), and Ommaya reservoir extraction (1/45). Among these cases, paired tumor tissue was obtained from 36 patients for gene sequencing. Both the CSF samples and paired tumor samples were sequenced using a panel targeting 131 genes (Fig. 1, Supplementary Table 1). We also summarized all cases’ clinical and molecular pathological characteristics (Supplementary Table 2). The median age at diagnosis for all patients was 39 years (range: 4–75 years). Pathological diagnoses included diffuse midline glioma (DMG), H3K27-altered (*n* = 14), glioblastoma (*n* = 1), H3-wildtype-astrocytoma (*n* = 10), ependymomas (*n* = 11), glioneuronal tumors (*n* = 3), and non-neoplastic lesions (n = 6). Most tumors were located in the cervical spinal cord (*n* = 20) and thoracic spinal cord (*n* = 14), with others in the cervicothoracic spinal cord (*n* = 8), thoracolumbar spinal cord (*n* = 1), and throughout the entire spinal cord (*n* = 2). The median tumor length was 4 segments (range: 1–9 segments). Additionally, 38 patients were newly diagnosed, while 7 had recurrent tumors. A total of 6 patients were diagnosed with a disseminated phenotype based on the magnetic resonance imaging (MRI) findings, with 5 of them exhibiting tumor dissemination at the time of diagnosis. Enhanced MRI showed intensification in 34 patients, while 11 patients had non-enhancing lesions.

### Higher rate of circulating tumor DNA positivity in cerebrospinal fluid among diffused midline glioma, H3K27-altered cases

**Fig. 2:**
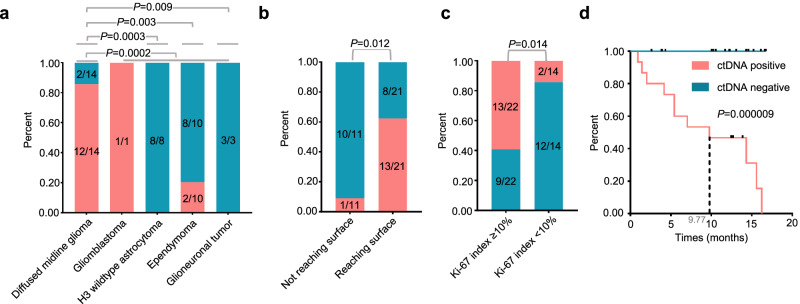
The proportion of ctDNA positive rate detected in CSF of patients with different lesions. **a** The proportion of ctDNA positive in CSF of cases with DMG-H3K27-altered and other tumors. **b** The proportion of patients with tumors reaching the surface of the spinal cord or not. **c** The proportion of ctDNA positive in CSF of cases with Ki-67 index ≥10% or <10%. **d** The overall survival of patients with positive ctDNA and negative ctDNA in CSF. Scale bar represents 1 month.

The median volume of CSF samples collected was 6.0 ml (range: 1.5–10.0 ml). Out of the samples, 5 had cfDNA concentrations below the low-detection limit (cfDNA < 10^−^^5^ ng/ml), including 2 samples from ependymoma patients and 3 from H3-wildtype-astrocytoma patients. Totally, we identified 52 mutations in the CSF cfDNA samples through targeted sequencing, with at least one genetic alteration detected in 51.1% (23/45) of samples from patients. None of alteration was identified in non-neoplastic lesions. We excluded cases with unclear external factors that they were without paired tissue and could not be identified as ctDNA positive, including two patients with H3-wildtype-astrocytoma, one with ependymoma and six with non-neoplastic lesion (Supplementary Fig. 2). Among the remaining 36 cases, 15 were classified as ctDNA positive. Subsequently, we examined various clinicopathological features of cases and their impact on rate of ctDNA positive in CSF samples (Table 1). Among these factors, tumors diagnosed with diffused midline glioma (DMG), H3K27-altered tumor (12/14 vs 3/22, *P* = 0.0002), reaching the surface of the spinal cord in MRI imaging (13/21 vs 1/11, *P* = 0.012), and Ki-67 index >=10% (13/22 vs 2/14, *P* = 0.014) showed higher rate of ctDNA positivity compared to others (Fig. 2a–c). Tumors with dissemination (4/6 vs 11/30, *P* = 0.210) and lesions showing enhancement (15/32 vs 0/4, *P* = 0.125) displayed a trend to higher positive rate of ctDNA (Supplementary Fig. 1a, b). A higher rate of ctDNA positive (6/6 vs 9/30, *P* = 0.00257) of cfDNA was observed in tumors with histological grade IV (Supplementary Fig. 1c). Patients with positive CSF ctDNA also exhibited significantly shorter overall survival [9.77 (0.78–18.76) months] compared to those with negative CSF ctDNA (undefined, *P* = 0.000009, Fig. 2d) (not including non-neoplastic lesions). We also observed that patients with positive ctDNA exhibited higher cfDNA concentration compared to those with negative ctDNA (Supplementary Fig. 1d, *P* = 0.00007). These findings suggest that ctDNA from spinal cord tumors with more aggressive features tend to be more frequently released into CSF.

**Table 1:** The impact of clinicopathological features on the ctDNA positivity

Patient characteristics	ctDNA positive (*n* = 36*)	*P* value
*Sex*		1.000
Male	6/14	
Female	9/22	
*Age*		0.443
<20	5/9	
≥20	10/27	
*Disease status*		0.677
Newly diagnosed	12/30	
Recurrent	3/6	
*Location of lesions*		0.082
C	8/17	
C-T	0/6	
T	5/10	
T-L	0/1	
Whole	2/2	
*Length*		0.734
<4 segment	6/17	
≥4 segment	9/19	
*Enhancement*		0.125
No	0/4	
Yes	15/32	
*Dissemination*		0.210
No	11/30	
Yes	4/6	
*Method of CSF extraction*		0.242
Lumber Puncture	14/33	
During surgery	0/2	
Ommaya Sac	1/1	
*Pathological diagnosis*		<0.001
Diffused midline glioma, H3K27-altered	12/14	
Astrocytoma	0/8	
Ependymoma	2/10	
Glioneuronal tumor	0/3	
Glioblastoma	1/1	
*H3K27M*		<0.001
No	3/22	
Yes	12/14	
*Ki-67 index*		0.014
<10%	2/14	
≥10%	13/22	

* 36 of 45 cases with sequencing data of paired tumor tissues.

### High concordance of mutations between cerebrospinal fluid circulating free DNA and tumor tissue in diffused midline glioma, H3K27-altered cases

**Fig. 3:**
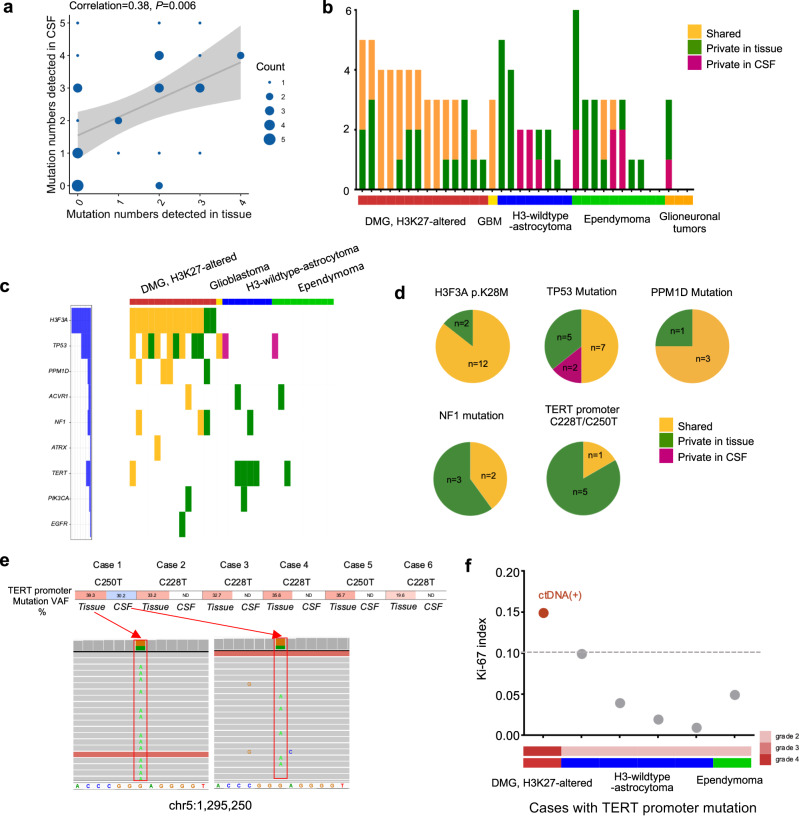
Mutations in paired CSF and tissue samples. **a** The correlation between mutation numbers detected in CSF and tissues. **b** The number of shared mutations, mutations private in tissue, and mutations private in CSF of each sample. **c** Mutations of featured genes in each sample. **d** The proportion of shared mutations, mutations private in tissue, and mutations private in CSF of key molecular features. **e** TERT promoter mutations in different types of tumor. **f** The Ki-67 index and histological grade of cases with TERT promoter mutation, the cases whose TERT promoter mutation was detected in CSF were labeled red, and the cases with tumor dissemination were labeled gray.

To evaluate the concordance between CSF and paired tumor tissue samples, we compared the mutations identified in both sample types. We observed a significant positive correlation (Pearson correlation = 0.38, *P* = 0.006) between the number of mutations detected in CSF and their corresponding tissue samples (Fig. 3a). Among the mutations identified, 37 were found in both the CSF and tissue samples, referred to as shared mutations. Additionally, excluding 3 mutations detected in patients without paired tumor tissue, 12 mutations were detected exclusively in the CSF samples, while 46 mutations were exclusively found in the tissue samples (Supplementary Fig. 2 and Supplementary Table 3). The shared or private mutation numbers of each type of cases were illustrated in Fig. 3b. The positive rate of ctDNA were 85.7% (12/14) in DMG, H3K27-altered patients, and 20.0% (2/10) in ependymoma patients. The glioblastoma case showed a complete set of paired mutations, while none of shared mutations were detected in all H3-wildtype-astrocytoma patients (0/8) and glioneuronal tumor patients (0/3). We also analyzed the variant allele frequency (VAF) of shared mutations and found that it was slightly higher (*P* = 0.0099) in tissue samples compared to CSF samples (Supplementary Fig. 3a). Moreover, there was a positive correlation (Pearson correlation = 0.45, *P* = 0.005) between the VAF of shared mutations in CSF and their corresponding tissue samples (Supplementary Fig. 3b).

We depicted the mutational profile of well-known tumor-related somatic mutations, including H3F3A, EGFR, TP53, PPM1D, ACVR1, NF1, ATRX, TERT promoter, and PIK3CA, using a waterfall plot (Fig. 3c). Among these mutations, the H3F3A p.K28M mutation (H3K27M mutation) displayed a strong concordance between the two sample types (Fig. 3d). However, there were two cases in which the H3F3A p.K28M mutation was not detected in the CSF but was present in the corresponding tumor tissues. In one of these cases, other mutations such as PPM1D and NF1, which were identified in the tumor tissue samples, were also not detected in the CSF samples (Supplementary Fig. 4a). Regarding TP53 mutations, the six shared mutations were exclusively observed in DMG, H3K27-altered (Supplementary Fig. 4b). Similarly, for the six cases with TERT promoter mutant tumors, we observed the presence of the TERT promoter mutation solely in the cerebrospinal fluid (CSF) of a case with DMG, while it was not detected in the CSF of the three H3-wildtype-astrocytomas or two ependymomas (Fig. 3c–e). The reads supporting TERT promoter mutations in tumors and CSF cfDNA were also showed Fig. 3e and Supplementary Fig. 4d. Furthermore, we observed that the case with positive TERT promoter mutation in the CSF displayed positive ctDNA, higher histological grade, and higher Ki-67 index compared to the remaining 5 cases which with negative ctDNA (Fig. 3f). Interestingly, the two cases with positive H3K27M-mutant tumors but negative H3K27M-mutant CSF cfDNA also displayed lower Ki-67 indexes and better survival compared to the remaining cases with the H3K27M mutation (Supplementary Fig. 4c). These findings suggested that the presence of mutations in the CSF may be associated with the increased malignancy of tumors.

### Share CNVs between CSF cfDNA and tumor tissue were exclusively observed in DMG, H3K27-altered cases

**Fig. 4:**
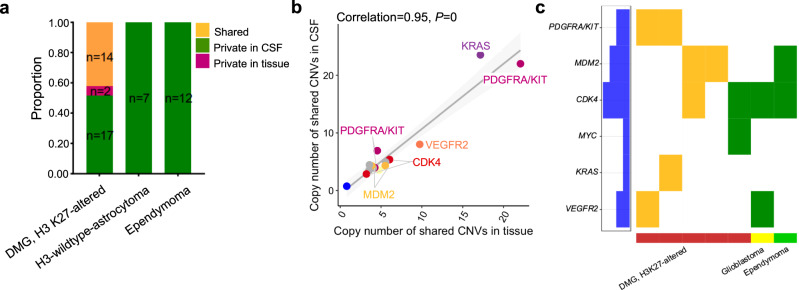
Copy number variations in paired CSF and tissue samples. **a** The proportion of shared CNV, CNV private in tissue, and CNV private in CSF of patients with different tumor types. **b** The correlation of copy number values of shared CNVs detected in CSF and tissues. **c** CNVs of selected genes in CSF and tissues. *P* and correlation value were calculated by Pearson correlation analysis.

In addition to mutation detection, we also conducted an analysis of CNVs in the genes included in our panel. Overall, we detected a total of 52 CNVs in the CSF and/or paired tumor tissue, with 14 shared CNVs, 2 CNVs private in the CSF samples, and 36 CNVs private in tumor tissue samples (Fig. 4a, Supplementary Fig. 5a and Supplementary Table 4). Notably, all shared CNV sites were exclusively detected DMG, H3K27-altered (Fig. 4a and Supplementary Fig. 5a).

The copy numbers of shared CNVs, such as PDGFRA/KIT, CDK4, MYC, KRAS, and VEGFR2, were highly consistent (Pearson correlation = 0.95, *P* = 0) between the CSF and tumor tissues (Fig. 4b). We also depicted the profiles of these CNVs as a waterfall plot (Fig. 4c and Supplementary Fig. 5b). These results suggest that the detection of PDGFRA/KIT amplification in CSF has the potential to predict the DMG, H3K27-altered.

### H3-K27M mutations in CSF as 100% specific predictors for DMG, H3K27-altered

**Fig. 5:**
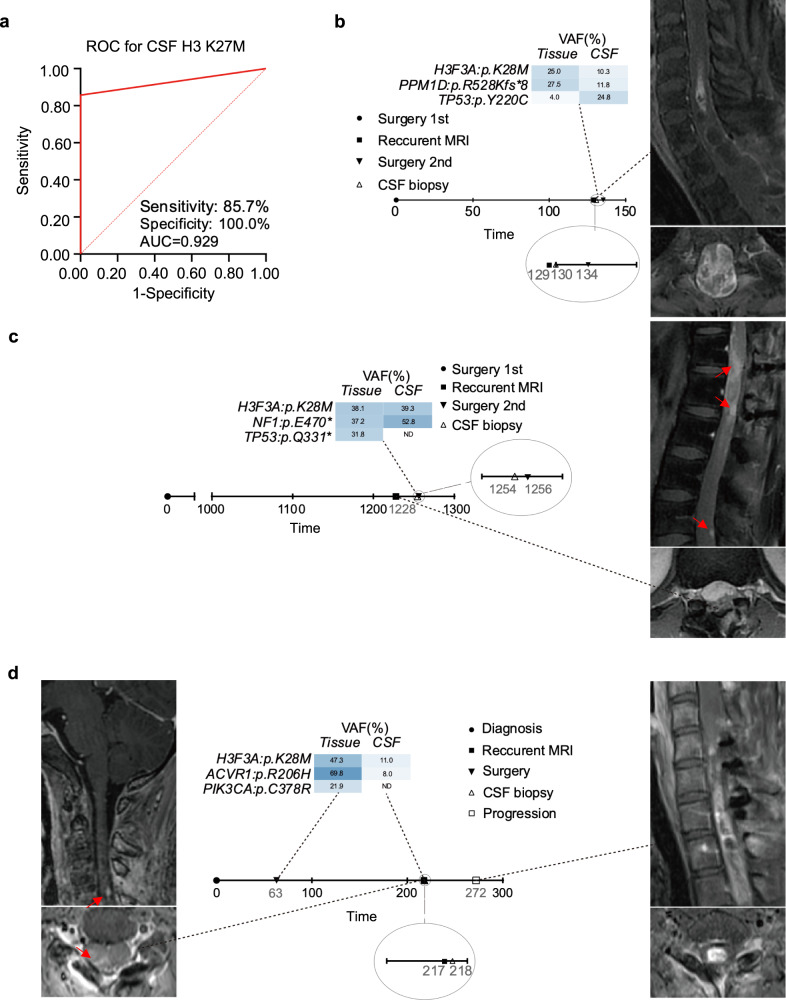
The performance of CSF cfDNA features in predicting DMG, H3 K27-altered. **a** The ROC curve for predicting DMG, H3 K27-altered via H3-K27M mutation in CSF. **b**–**d** Detecting of H3F3A p.K28M mutations in CSF of recurrent H3 K27M-mutant tumors. Scale bar represents 1 day.

Accurate diagnosis and differentiation of DMG, H3K27-altered from other intramedullary tumors are essential for determining appropriate treatment and assessing prognosis. Here, we found that the detection of DMG driver mutation, H3K27M, in the cerebrospinal fluid (CSF) of patients can be utilized for pre-surgery differential diagnosis of DMG, H3K27-altered, with a sensitivity of 85.7%, specificity of 100%, and AUC of 0.929 (Fig. 5a). These results underscore the utility of quantifying and detecting key molecular alterations in the pre-surgery differential diagnosis of DMG, specifically H3K27-altered.

### Potential of H3K27M mutation in monitoring DMG, H3K27-altered tumor recurrence

We also observed the presence of H3K27M mutations in the CSF samples of three patients who experienced tumor relapse after the initial surgery (Fig. 5b–d). Among them, case 1 and case 2 had undergone the initial surgery at other hospitals before seeking treatment at our institution due to the recurrence of symptoms. To confirm the tumor recurrence, these patients underwent MRI imaging examinations, and subsequently, CSF cfDNA sequencing was performed. The sequencing results revealed specific mutations in each case. In case 1, the sequencing identified H3F3A: p.K28M, PPM1D: p.R528Kfs*8, and TP53: p.Y220Cmutations (Fig. 5b), while in case 2, the detected mutations were H3F3A: p.K28M and NF1: p.E470* (Fig. 5c). These findings provide additional evidence of the recurrence of H3K27M-mutant tumors.

In the case 3, the initial surgery was conducted at our hospital, and post-surgery treatments were administered following the Stupp protocol typically used for brain glioblastoma. While undergoing adjuvant therapy, a suspicious disease relapse was detected during routine MRI imaging. In response, the patient promptly considered a change in the treatment regimen to third-line intrathecal chemotherapy and provided CSF samples for further confirmation of the disease status. Unfortunately, the CSF analysis revealed the presence of H3F3A: p.K28M and ACVR1: p.R206H mutations, which were consistent with the mutations identified in the primary tumor resected during the initial surgery. Subsequently, after a span of 54 days, the patient’s condition deteriorated rapidly, and disseminated recurrences were confirmed (Fig. 5d). These findings support for the potential utility of CSF detection as a means to monitor tumor relapse in DMG cases.

## Discussion

The pre-surgery differential diagnosis of intramedullary gliomas, particularly those with H3K27M alterations, remains challenging due to the limited imaging characteristics of these lesions. Additionally, performing a biopsy on the spinal cord is a highly invasive and high-risk procedure that can result in unnecessary neurological impairment^16–18^. To address these issues, the utilization of less-invasive cerebrospinal fluid (CSF)-derived cell-free DNA (cfDNA) offers unique advantages in assessing the molecular characteristics of central nervous system (CNS) tumors^19,20^. Here, using a developed panel for next-generation sequencing (NGS), we observed highest percentages of ctDNA positive cases in patients with diffuse midline glioma (DMG), H3K27-altered, compared to other intramedullary tumor types. Moreover, the shared mutations between CSF and paired tumor tissues were more readily detected in DMG, H3K27-altered. Furthermore, we found that our CSF detection exhibited a high detection rate of 85.7% (12/14) and 100% specificity for DMG, H3K27-altered. The detection of cfDNA mutation could give a chance to replace biopsies of tumor tissue, contributing to the development of noninvasive monitoring for recurrent patients^21,22^. Collectively, these findings highlight the feasibility of CSF cfDNA sequencing, shedding light on the potential accuracy of CSF cfDNA detection in the differential diagnosis of patients with DMG, H3K27-altered subgroup.

Our results also demonstrate that prediction can be initiated by employing a well-established capture-based targeted sequencing panel when suspicious lesions were considered in early screens. This approach facilitates the generalization and application of molecular diagnosis for specific intramedullary spinal cord without additional cost or optimization of panel design. In comparison to panel sequencing, digital droplet polymerase chain reaction (ddPCR) is more cost and time-effective, while next-generation sequencing (NGS) offers an advantage of collecting more extensive information relative to lower-throughput sequencing^23^. However, it is worth noting that ddPCR or Nanopore sequencing requires specific equipment and may not be widely adopted in molecular testing departments of neuropathology. In contrast, panel target sequencing has been extensively employed in the testing of tumor tissue and can offer a viable option for a larger number of patients.

Our study revealed that there was a higher positive rate of ctDNA in DMG, H3K27-altered paitents, compared to patients H3-wildtype-astrocytomas, ependymomas and glioneuronal tumors. Although low-grade tumors have fewer somatic mutations^24^, less release of ctDNA in these tumors with lower degree of malignancy is noteworthy. Additionally, we demonstrated that ctDNA positivity and H3K27M mutations were not detected in two cases of H3K27M-mutant tumors, which exhibited less aggressive clinicopathological features such as a low ki-67 index, absence of enhancement on MRI images, and relatively better prognosis. Similarly, TERT promoter mutation, which has been reported to be associated with a worse prognosis in spinal cord astrocytoma^25^, was only detected in CSF of one DMG case, which exhibited more aggressive clinicopathological features. Again, these findings corroborate that molecular profiling using CSF cfDNA detection is feasible for tumors with high malignancy. Interestingly, our previous researched revealed histological grades have significant impact on the prognosis of DMG, H3K27-altered patients, indicating more studies are required to uncover the heterogeneity of these tumors with same driver mutations^3,7^.

In comparison, profiling the molecular characteristics of H3-wildtype-astrocytomas and ependymomas through CSF detection remains challenging, including the identification of specific driver mutations like TERT promoter mutations. Our finding not only supports and extends a preliminary study by Ian et al., which reported unreliable detection of ctDNA in the CSF of their ependymoma cohort^26^, but also agreed with previous reports about the feasibility of TERT promoter alteration detection in glioblastoma patients^27,28^. We speculated that tumors with higher histological grades exhibit more aggressiveness and metastatic capacity, releasing more ctDNA. Conversely, in the remaining five tumor cases with lower malignancy, rare tumor DNA was released from tumor in CSF, causing difficulty in detecting any alterations. In this setting, employing more sensitive detection methods, such as ddPCR, may prove beneficial in handling lower quantity of target fragment of DNA^29,30^. However, further research using more precise methods is warranted to verify the sequencing efficiency.

To date, there have been limited reports regarding the detection of copy number variations (CNVs) using CSF cfDNA^31,32^. Here, we successfully observed shared CNVs between CSF cfDNA and tumor tissue DNA in cases with DMG, H3K27-altered but not in other tumor types. Additionally, these shared CNVs are mainly well-known CNVs in DMG, H3K27-altered, such as PDGFRA amplification, MDM2 amplification, and others^33^. Moreover, we demonstrated that the copy numbers of these shared mutations were highly consistent between the CSF cfDNA and tumor tissues, just as previously reported in medulloblastoma^34^. All of these findings demonstrated the feasibility of our panel sequencing in detecting CNVs in CSF cfDNA. High-throughput, rapid screening like Nanopore might offer an instant CNV spectrum^31^. However, considering the sample size in our study may have been relatively small, which could impact the generalizability of the findings. Larger cohorts are needed to validate and further explore the utility of CSF cfDNA for CNV detection.

Our study had several limitations. Firstly, the follow-up period was limited, and we did not collect CSF samples for the purpose of dynamic monitoring of cfDNA. Conducting longitudinal evaluations would provide a more comprehensive understanding of how cfDNA levels change over time and their correlation with disease progression and treatment response. Furthermore, while our study enrolled a relatively large number of cases of spinal cord tumors overall, the number of individuals within each specific tumor type was still limited. Due to the disease incidence and willingness of patients to enroll, there exists an imbalanced distribution of astrocytoma and ependymoma in our cohort. Increasing the cohort size for each tumor type would enhance the statistical power and robustness of our findings.

In conclusion, our findings contribute to the understanding of the role of CSF cfDNA in spinal cord tumors and highlight the potential of cfDNA analysis for molecular profiling and disease characterization. Our study demonstrated the feasibility of molecular profiling of CSF cfDNA in the differential diagnosis of DMG, H3K27-altered. However, our study also revealed challenges in detecting tumor cfDNA in the CSF of H3-wildtype-astrocytomas and ependymomas.

## Methods

Patients with spinal cord lesions (*n* = 45) were enrolled in this prospective study between August 2021 and October 2023 in the Department of Neurosurgery of Beijing Tiantan Hospital. This study protocol followed the principles of the Declaration of Helsinki and was approved by the Institutional Review Board and Ethics Committee of Beijing Tiantan Hospital (Beijing, China, IRB: KY 2020-023-03). For the export of human genetic data, our study has received formal approval from the Guidance of the Ministry of Science and Technology (MOST) for the Review and Approval of Human Genetic Resources. Each patient participating in our research provided written informed consent for the utilization of clinical data, photographs, and samples. MRI scans were performed on all patients. Cerebrospinal fluid (CSF) samples (1.5–10.0 ml) were collected before surgery from 45 patients via preoperative lumbar puncture (41/45), intraoperative extraction (3/45), and Ommaya reservoir extraction (1/45) (Fig. 1). Tissue of lesions was obtained during surgical resection or biopsy for 36 patients in the study. After surgery, the tissue’s pathologic results were retrospectively reviewed with the final pathology findings as determined.

### Circulating cell-free DNA (cfDNA) isolation from cerebrospinal fluid (CSF)

EDTA tubes containing CSF (1.5–10.0 ml) were centrifuged for 10 min at 1200 × *g*, and the supernatants were further centrifuged for 10 min at 12,000 × *g*. Then, circulating nucleic acid was extracted from CSF using the Apostle MiniMax High-Efficiency cfDNA Isolation Kit (APOSTLE, USA) following the manufacturer’s instructions. cfDNA eluted in 20 μL AVE buffer (QIAamp Circulating Nucleic Acid Kit) or in 20 μL nuclease-free water (ExoLution PLUS Kit) and stored at −20 °C.

### DNA isolation from tissue

DNA was isolated from formalin-fixed and paraffin-embedded tumor tissue using the QIAamp DNA Tissue & Blood Kit (Qiagen, Cat, Germany) as recommended by the manufacturer. DNA was eluted in AE buffer (Qiagen) and stored at −20 °C until further processing.

### NGS library preparation

The purified cfDNA and tissue DNA were quantified by the Qubit dsDNA High Sensitivity Assay kit and Qubit fluorometer 4.0 (Life Technologies, USA). The genomic DNA libraries were constructed with the KAPA HyperPlus Kit (Roche, Swiss). The condition for enzymatic fragmentation reaction time was optimized to 17 min. All CSF cfDNA was used for library construction using the xGen^TM^ Prism DNA Library Prep Kit (IDT, USA) and further used to construct hybridization libraries with the KAPA Hyper Capture Reagent Kit (Roche, Swiss). All DNA libraries were captured with a designed panel of 131 brain tumor-related genes and sequenced by Simcere Diagnostics, Inc. (Nanjing, China).

### Sequencing data procession

A custom pipeline was developed to perform reads alignment, variants calling, fusion detection, CNV identification, and quality control. The software package fastp (v.2.20.0) was used for adapter trimming and quality trimming^35^. Sequence reads were aligned against the human reference genome (hg19) using BWA-mem (v.0.7.17)^36^. VarDict (v.1.5.7) was used for SNVs and Indels detection, while the CNVkit (dx1.1) was used for chromosome (arm) CNVs processing^37,38^. Filtering was performed based on the following criteria. For SNVs, the threshold we adopted for retaining a mutation in CSF and tissue was that it had a total of ≥4 reads for general genes and ≥2 reads for hotspot genes, and a VAF greater or equal to 10% in tissue and 5% in CSF. When either mutation is present in both samples, the VAF threshold loosens to 1%. For germline mutations, to choose only heterozygous variants, we focused on 0.20 < = VAF < = 0.80, and mutations in the control cohort of the National Center for Biotechnology Information (NCBI, https://www.ncbi.nlm.nih.gov/) and cBioPortal (https://www.cbioportal.org/) database were manually reviewed and removed to ensure that polymorphism was not present in the final set of mutations. For CNVs, only regions which included >=5 probes were considered. Segments with copy numbers above 3 or below 1 were considered as amplifications or deletions.

### Data analysis and visualization

Statistical analysis was performed using R (v4.2.2) or GraphPad Prism software version 8.0. The Fisher exact test (or chi-squared test when appropriate) was used for all categorical variables. We employed a non-parametric test due to the uneven number of participants in two groups and the non-normal data distribution. The center line represents the median and error bars represent range of scatter. Pearson and Spearman test was used to correlate coefficients among outcomes of continuous and categorical variables, respectively. Specificity and sensitivity were derived from areas under the ROC curves (AUC-ROC). *P* values were reported as 2-sided, with statistical significance defined as *P* < 0.05.

### Reporting summary

Further information on research design is available in the Nature Research Reporting Summary linked to this article.

### Supplementary information

Supplementary Figure 1-5, Supplementary Table 1-4

REPORTING SUMMARY

## Data Availability

The raw sequence data reported in this paper have been deposited in the Genome Sequence Archive (Genomics, Proteomics & Bioinformatics 2021) in National Genomics Data Center (Nucleic Acids Res 2022), China National Center for Bioinformation/Beijing Institute of Genomics, Chinese Academy of Sciences (GSA-Human: HRA006191) that are publicly accessible at https://ngdc.cncb.ac.cn/gsa-human^39,40^. The datasets used and/or analyzed during the current study available from the corresponding author on reasonable request. All data discussed in this manuscript are included in the main paper text and the Supplementary Materials.

## Code availability

The sequencing data was analyzed using fastp (v.2.20.0), BWA-mem (v.0.7.17), VarDict (v.1.5.7) and CNVkit (dx1.1) packages. Statistical analyses were performed in R version 4.2.2 or GraphPad Prism version 8.0. No custom code was used in this study. The detailed analysis code used in this paper is available upon request to the corresponding authors.
