# Supplementary material for: Sequencing of cerebrospinal fluid cell-free DNA facilitated early differential diagnosis of intramedullary spinal cord tumors
Source: NPJ Precis Oncol. 2024 Feb 22;8:43. doi: 10.1038/s41698-024-00541-w (PMC10884012; doi:10.1038/s41698-024-00541-w)
Supplement: Supplementary file 1 — Supplementary Figure 1-5, Supplementary Table 1-4 [file 41698_2024_541_MOESM1_ESM.pdf]

Supplementary Figure 1

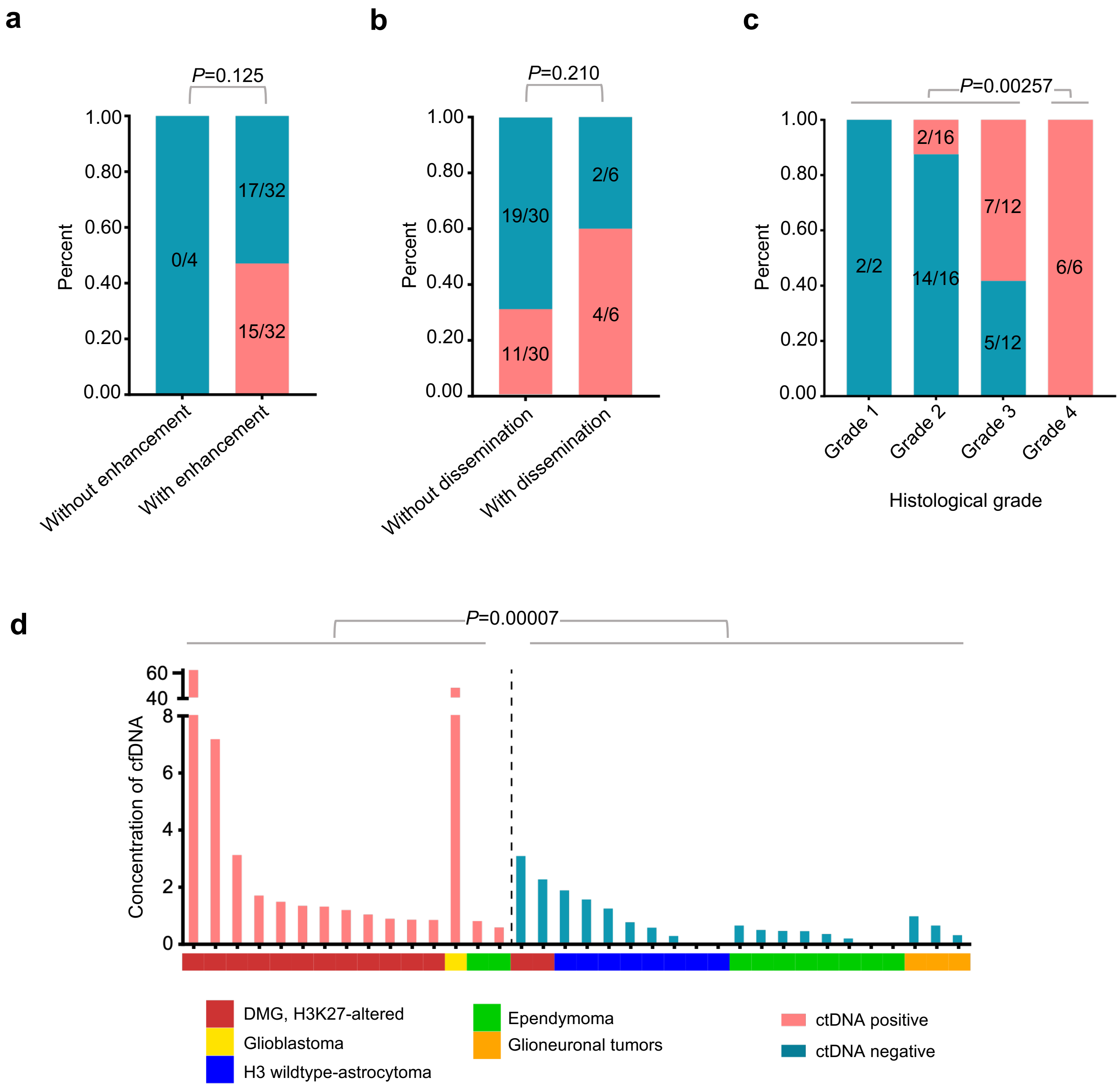

Supplementary Figure 1. Proportion of ctDNA positive in CSF of patients with different lesions. a The proportion of patients with or without enhancement. **b** The proportion of patients with or without dissemination. **c** The proportion of ctDNA positive in CSF of cases with different histological grade tumors. **d** ctDNA status and concentration of cfDNA in each sample of tumor. The concentration of cfDNA in other cases, including cases without paired tissues n=3 or non-neoplastic lesions n=6 was found in Supplementary Table 2. Scale bar represents 1ng/ml.

Supplementary Figure 2

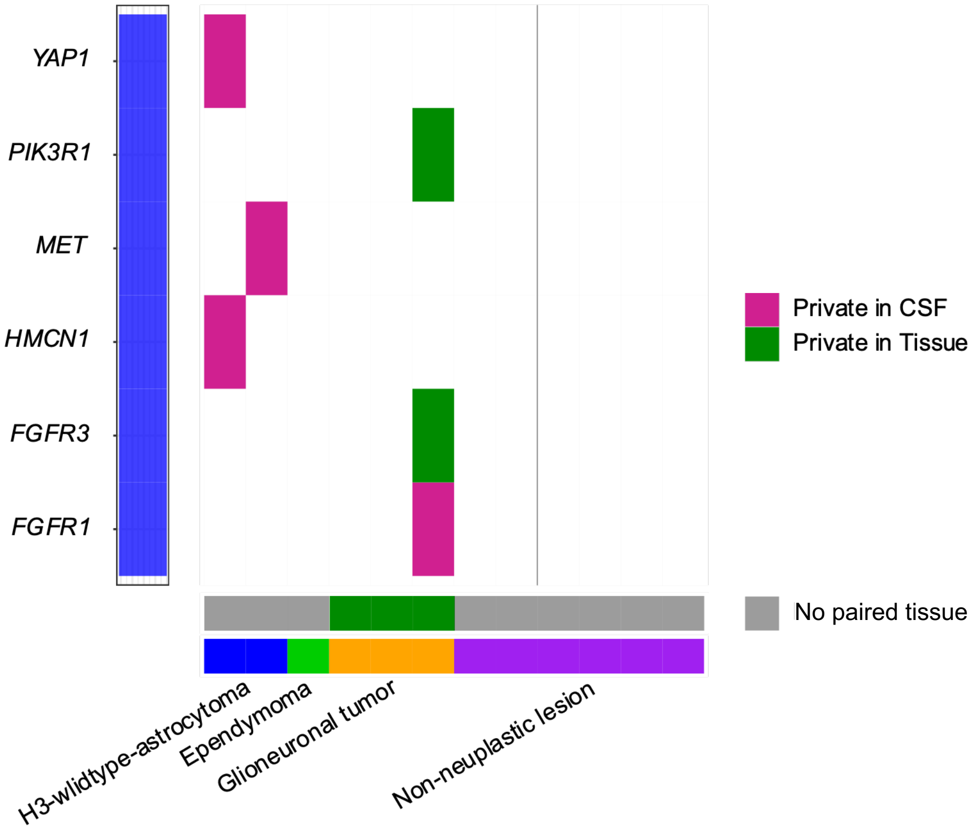

Supplementary Figure 2. Landscape of mutations in paired CSF and tissue of each rest patients. None of shared mutation were detected in 3 patients with glioneuronal tumor. Three mutations were detected in CSF samples from 2 patients with H3-wildtype astrocytoma, but none of mutations were detected in CSF from 1 patient with ependymoma and 6 patients with non-neoplastic lesion.

Supplementary Figure 3

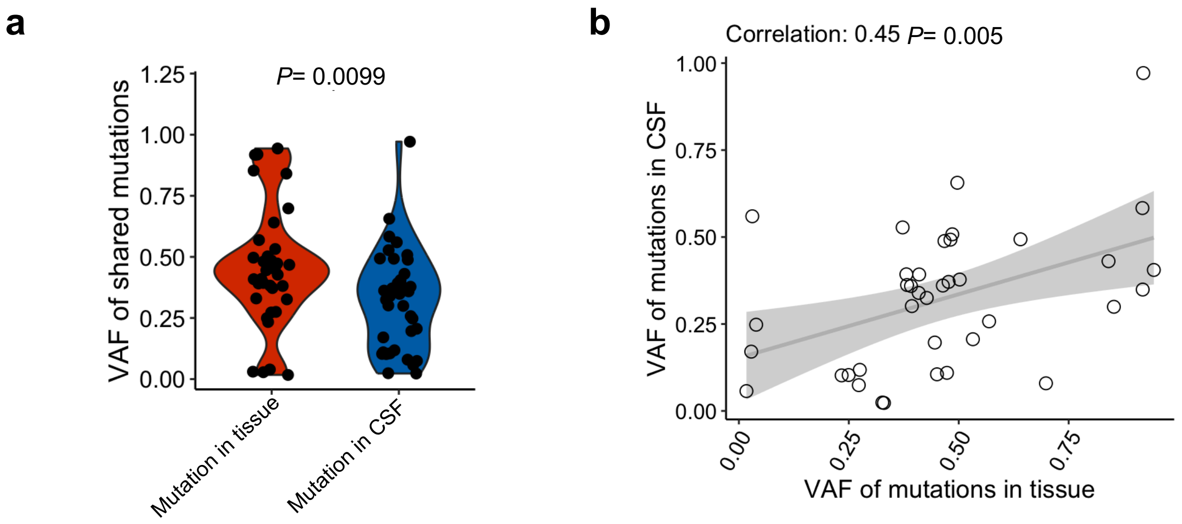

Supplementary Figure 3. VAF of mutations detected in tumor tissues and CSF cfDNA. a The VAF values of shared mutations in CSF and tissue. **b** Correlation between VAF of mutations in CSF and tissue.

Supplementary Figure 4

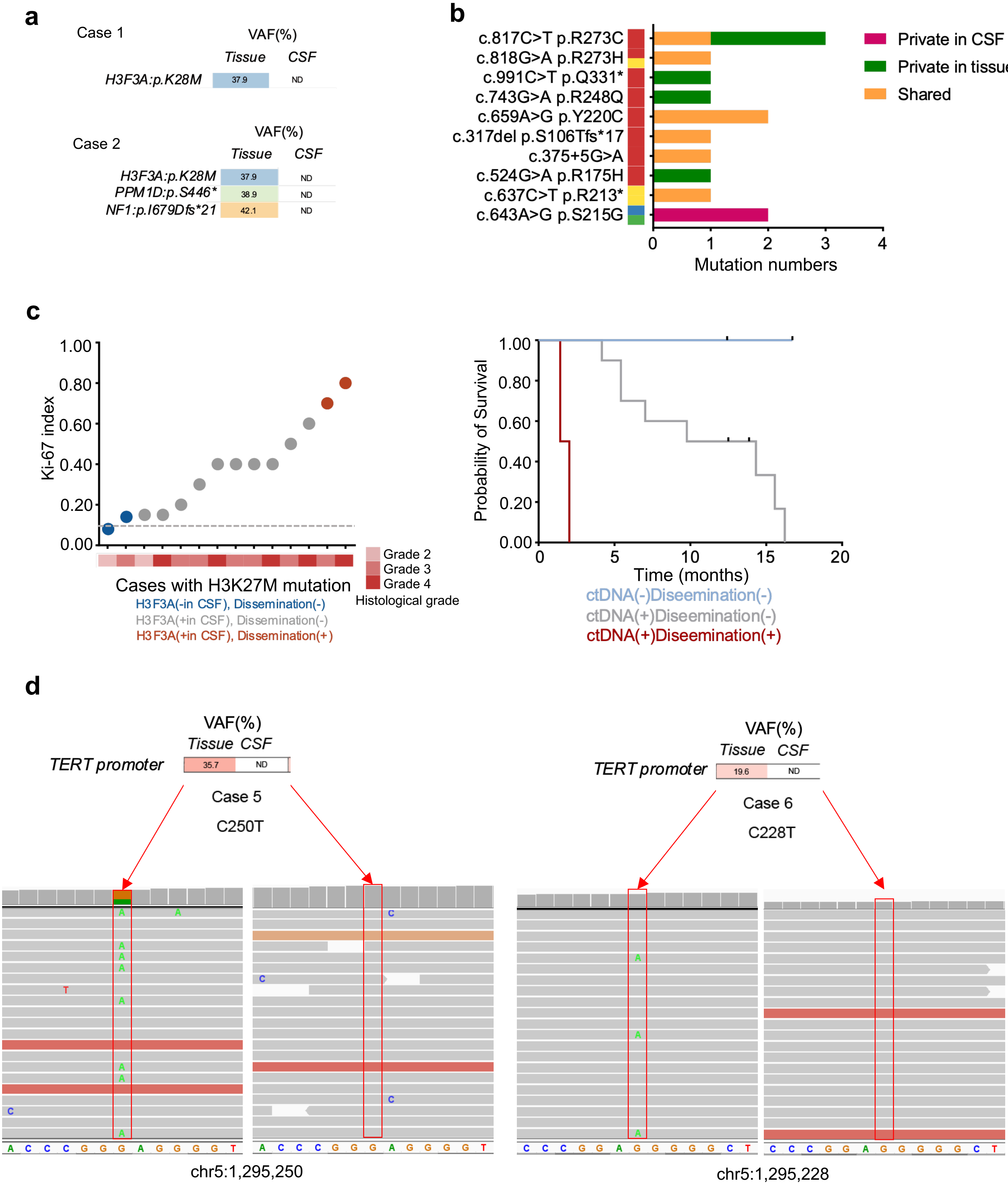

Supplementary Figure 4. Cases whose key molecular features were not detected in CSF. a mutations detected in tissue and CSF of H3 K27M-mutant cases whose H3 K27M were not detected in CSF samples. **b** TP53mutations in CSF and tumor tissues. **c** The Ki-67 index and survival curves of cases with DMG, H3K27-altered, the cases whose H3K27M mutation was not detected in CSF were labeled blue, and the cases with tumor dissemination were labeled red. **d** representative examples from TERT promoter mutant patients with H3-wildtype astrocytoma and ependymoma.

Supplementary Figure 5

a

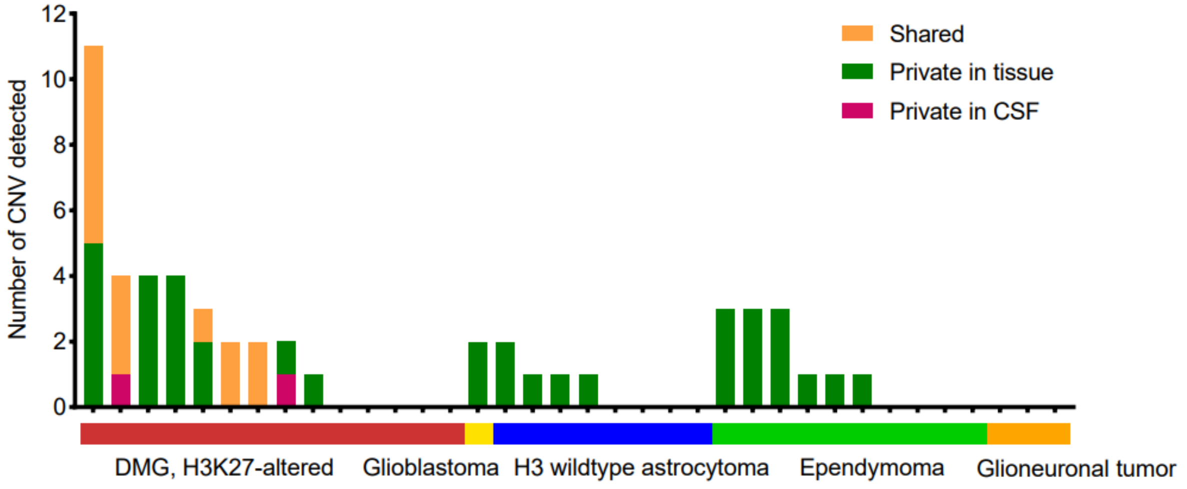

b

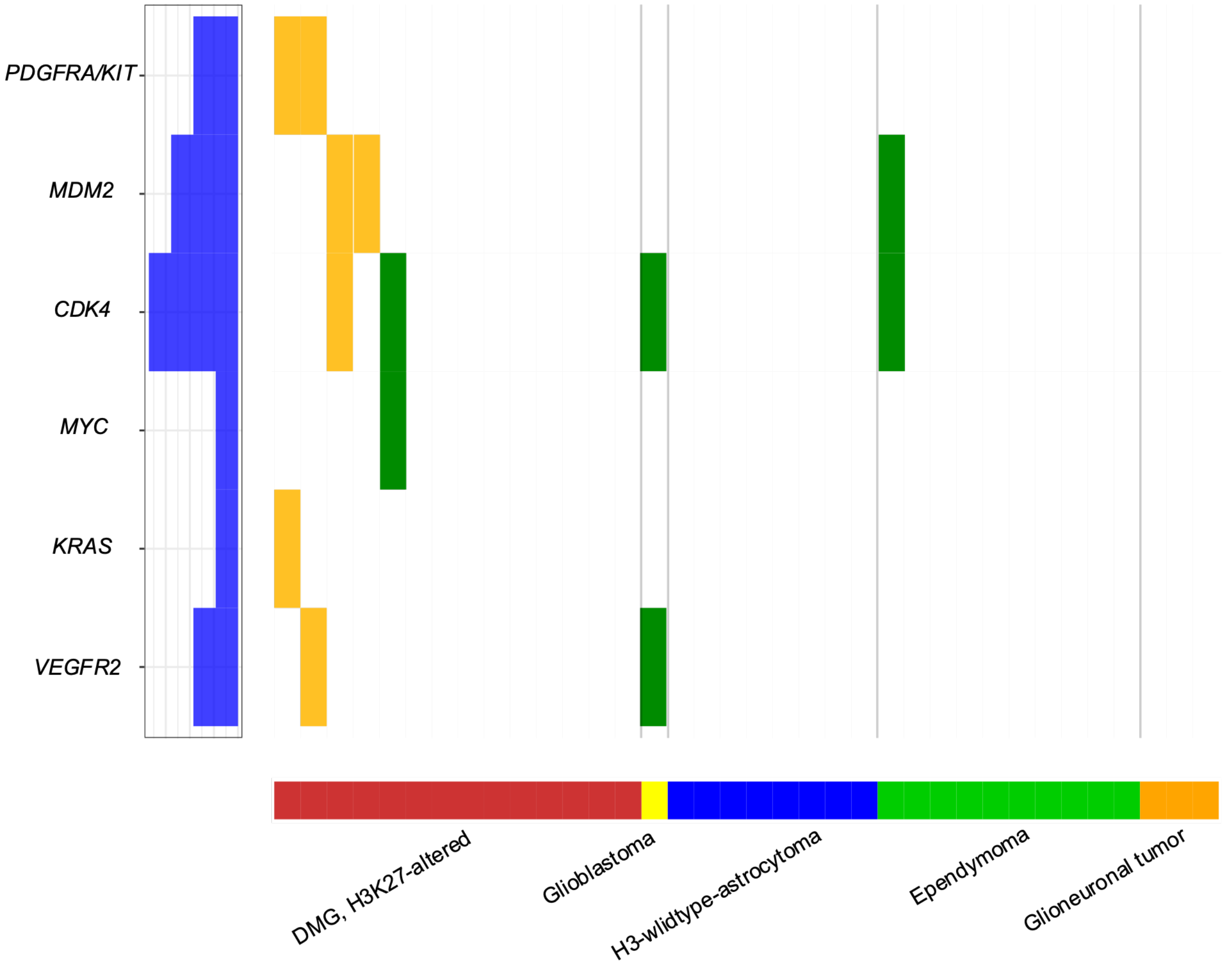

Supplementary Figure 5. Copy number variants CNV in each patients. a CNV detected in CSF cfDNA and tumor tissue DNA of each patients. **b** The landscape of selected CNVs.

Supplementary table1. The list of target genes sequenced.

ACVR1	AKT1	AKT2	ALK	APC	AR	ARAF	ARID2	ATM
ATRX	B2M	BCL2L11	BCOR	BRAF	BRCA1	BRCA2	CBL	CCND2
CDK4	CDK6	CDKN2A	CDKN2B	CDKN2C	CHEK2	CIC	CTNNB1	DAXX
DDR2	DDX3X	DICER	DNMT3A	EGFR	EPCAM	ERBB2	ERBB3	ERBB4
ESR1	FAT1	FBXW7	FGF4	FGFR1	FGFR2	FGFR3	FGFR4	FLT3
FLT4	FUBP1	GNA11	GNAQ	GNAS	H3F3A	HDAC4	HIST1H3B	HIST1H3C
HMCN1	HNF1A	HRAS	IDH1	IDH2	IRS2	JAK1	JAK2	KDM5A
KIT	KLF4	KRAS	MAP2K1	MAPK1	MDM2	MDM4	MEN1	MET
MLH1	MPL	MSH2	MSH6	MTOR	MYB	MYC	MYCN	NAB2
NF1	NF2	NOTCH1	NR3C1	NRAS	NTRK1	NTRK2	NTRK3	PDGFRA
PDGFRB	PIK3CA	PIK3CB	PIK3R1	PLCG1	PMS2	POLE	POLR2A	PPM1D
PTCH1	PTEN	PTPN11	RAF1	RB1	RELA	RET	RGPD3	RICTOR
ROS1	SDHA	SETD2	SMAD4	SMARCA4	SMARCB1	SMARCE1	SMO	SRC
STAG2	STAT6	TERT	TP53	TRAF7	TSC1	TSC2	USP8	VEGFA
VEGFB	VEGFR1	VEGFR2	VHL	YAP1				

Supplementary Table 2. Clinical and pathological features of patients.

Patient Characteristics (n=45)		
Sex		
	Male	27(60.0%)
	Female	18(40.0%)
Age (years)	Median（Range）	39(4-75)
Disease state		
	Primary	38(84.4%)
	Reccurent	7(15.6%)
Location of lesions		
	C	20(44.4%)
	C-T	8(17.8%)
	T	14(31.1%)
	T-L	1(2.2%)
	Whole	2(4.4%)
Length (segments)	Median（Range）	4(1-9)
Enhancement		
	No	11(24.4%)
	Yes	34(75.6%)
Dissemination		
	No	39(86.7%)
	Yes	6(13.3%)
Method of CSF extraction		
	Lumber puncture	41(91.1%)
	Ommaya Sac	1(2.2%)
	During surgery	3(6.7%)
Volume of CSF (ml)	Median（Range）	6.0(1.5-10.0)
Concentration of ctDNA (ng/ml)	Median（Range）	0.837(0.153-61.500)
Pathological diagnosis		
	Diffused Midline Gliomas,H3K27-altered	14(31.1%)
	H3-Wildtype Astrocytoma	10(22.2%)
	Ependymoma	11(24.4%)
	Glioneuronal Tumor	3(6.7%)
	Glioblastoma	1(2.2%)
	Non-neoplastic lesion	6(13.3%)
H3K27M mutation		
	No	31(68.9%)
	Yes	14(31.1%)
Histological grade		
	Non-neoplastic lesion	6(13.3%)
	1	3(6.7%)
	2	18(40.0%)
	3	12(26.7%)
	4	6(13.3%)
Ki-67 index (%)	Median（Range）	8(1-80)

Supplementary Table 3. Mutations identified in tumor tissue and cerebrospinal fluid.

Sample	Shareded situation	Chromosome	Position	Mutational type
2	Tissue only	chr1	226252135	SNV
3	Shared	chr1	226252135	SNV
3	Shared	chr4	55598066	SNV
3	Shared	chr17	29527590	SNV
3	Shared	chr17	58740509	SNV
4	Shared	chr1	226252135	SNV
4	Tissue only	chr19	42791286	SNV
4	Tissue only	chr1	186039779	SNV
4	Tissue only	chr9	5089776	SNV
4	Shared	chr17	7577538	SNV
5	Shared	chr4	187540610	SNV
5	Shared	chr13	28592686	SNV
5	Shared	chr1	226252135	SNV
5	Tissue only	chr17	7577121	SNV
6	Shared	chrX	76938752	SNV
6	Shared	chr1	226252135	SNV
6	Shared	chr11	64572612	SNV
6	Shared	chr5	1295250	SNV
7	Tissue only	chr1	226252135	SNV
7	Tissue only	chr20	39791337	SNV
7	Tissue only	chr17	29541484	smallINDEL
8	Shared	chr1	226252135	SNV
8	Shared	chr17	58740674	smallINDEL
8	Shared	chr17	7578206	SNV
9	Shared	chr1	226252135	SNV
9	Tissue only	chr17	7578206	SNV
11	Tissue only	chr7	55221822	SNV
11	Shared	chr1	226252135	SNV
11	Shared	chr17	7577121	SNV
11	Tissue only	chr12	56481361	SNV
12	Shared	chr2	158630626	SNV
12	Shared	chr1	226252135	SNV
12	Tissue only	chr3	178922363	SNV
13	Tissue only	chr17	37866383	SNV
13	Shared	chr9	21971029	SNV
13	Shared	chr1	226252135	SNV
13	Tissue only	chr17	7577121	SNV
14	Shared	chr17	29541484	SNV
14	Shared	chr1	226252135	SNV
14	Tissue only	chr17	7576855	SNV
15	Shared	chr17	58740521	smallINDEL
15	Shared	chr1	226252135	SNV
15	Shared	chr6	33286790	SNV
16	Shared	chr17	7579369	smallINDEL
16	Shared	chr5	1295250	SNV
16	Shared	chr1	226252135	SNV
16	Tissue only	chr2	107029692	SNV
16	Tissue only	chr17	7578406	SNV
17	Shared	chr17	7578212	SNV
17	Shared	chr17	7578190	SNV
17	Shared	chr11	119148919	SNV
18	CSF only	chr17	7578206	SNV
18	Tissue only	chr15	66782095	SNV
19				
20	Tissue only	chr2	158630626	SNV
20	Tissue only	chr12	25398284	SNV
20	Tissue only	chr3	12626634	SNV
20	Tissue only	chr7	128843429	SNV
20	Tissue only	chr5	1295228	SNV

21	Tissue only	chr3	178936092	SNV
21	Tissue only	chr5	1295228	SNV
22	Tissue only	chr4	187628565	SNV
22	Tissue only	chr17	29541484	SNV
22	Tissue only	chr17	29541484	SNV
22	Tissue only	chr5	1295228	SNV
23	CSF only	chr1	186039779	SNV
23	CSF only	chr16	2134509	SNV
24	CSF only	chr11	64572612	SNV
25	Tissue only	chr5	1295250	SNV
26	CSF only	chr7	128843429	SNV
26	CSF only	chr5	38953624	SNV
27	CSF only	chr10	43601830	SNV
27	CSF only	chrX	76938752	SNV
28				
29	Tissue only	chr16	2134509	SNV
29	Tissue only	chr14	105246551	SNV
29	Tissue only	chr5	1295228	SNV
30	Tissue only	chr22	29091840	MNV
30	Tissue only	chr19	11098450	SNV
30	Tissue only	chr3	12626634	Deletion
30	Tissue only	chr22	30051618	SNV
30	CSF only	chr12	56495026	SNV
30	CSF only	chr2	107049681	SNV
31	Tissue only	chr17	37872126	SNV
31	Tissue only	chr3	138417822	SNV
31	Tissue only	chr1	185969311	SNV
32	Tissue only	chr3	47164921	SNV
33				
34	Shared	chr2	29606696	SNV
34	Tissue only	chr17	37866383	SNV
34	Shared	chr13	28592686	SNV
35				
36	Tissue only	chr4	187628853	SNV
37	Tissue only	chr2	158630626	SNV
37	CSF only	chr11	119149251	SNV
37	CSF only	chr6	117622231	SNV
38	CSF only	chr17	7578206	SNV
38	Shared	chr17	41256183	SNV
38	CSF only	chr11	119170339	SNV
39				
40				
41	Tissue only	chr3	138417822	Deletion
41	Tissue only	chr4	187540610	SNV
41	CSF only	chr4	187540610	SNV
42				
43				
44				
45				
46				
47				

Hugo_Symbol	RefSeq	HGVSc	HGVSp	Depth in CSF
H3F3A	NM_002107.4	c.83A>T	p.K28M	Negative
H3F3A	NM_002107.4	c.83A>T	p.K28M	31.57368
KIT	NM_000222.2	c.2263G>A	p.A755T	34.49196
NF1	NM_000267.3	c.1039C>T	p.Q347*	37.84974
PPM1D	NM_003620.3	c.1414G>T	p.E472*	57.67119
H3F3A	NM_002107.4	c.83A>T	p.K28M	240.7176
CIC	NM_015125.4	c.346G>T	p.V116L	Negative
HMCN1	NM_031935.2	c.8029C>T	p.P2677S	Negative
JAK2	NM_004972.3	c.2674C>G	p.L892V	Negative
TP53	NM_000546.5	c.818G>A	p.R273H	261.2196
FAT1	NM_005245.3	c.7130C>T	p.T2377M	1124.8567
FLT3	NM_004119.2	c.2459C>A	p.T820N	2388.91854
H3F3A	NM_002107.4	c.83A>T	p.K28M	2492.49772
TP53	NM_000546.5	c.817C>T	p.R273C	Negative
ATRX	NM_000489.4	c.1996C>T	p.R666*	153.08514
H3F3A	NM_002107.4	c.83A>T	p.K28M	507.3762
MEN1	NM_130799.2	c.1244G>A	p.R415Q	589.22514
TP53	NM_198253.2	c.375+5G>A	.	715.9965
H3F3A	NM_002107.4	c.83A>T	p.K28M	Negative
PPM1D	NM_002660.2	c.1337C>G	p.S446*	Negative
NF1	NM_000267.3	c.2033dup	p.I679Dfs*21	Negative
H3F3A	NM_002107.4	c.83A>T	p.K28M	267.79492
PPM1D	NM_003620.3	c.1582dup	p.R528Kfs*8	306.16244
TP53	NM_000546.5	c.659A>G	p.Y220C	642.65596
H3F3A	NM_002107.4	c.83A>T	p.K28M	137.6865
TP53	NM_000546.5	c.743G>A	p.R248Q	Negative
EGFR	NM_005228.3	c.866C>T	p.A289V	Negative
H3F3A	NM_002107.4	c.83A>T	p.K28M	1259.9392
TP53	NM_000546.5	c.817C>T	p.R273C	2505.43488
ERBB3	NM_001982.3	c.548G>T	p.C183F	Negative
ACVR1	NM_001105.4	c.617G>A	p.R206H	111.7452
H3F3A	NM_002107.4	c.83A>T	p.K28M	154.05376
PIK3CA	NM_006218.3	c.1132T>C	p.C378R	Negative
ERBB2	NM_004448.3	c.688C>T	p.P230S	Negative
CDKN2A	NM_000077.4	c.329G>A	p.W110*	202.61596
H3F3A	NM_002107.4	c.83A>T	p.K28M	265.48256
TP53	NM_000546.5	c.817C>T	p.R273C	Negative
NF1	NM_000267.3	c.1408G>T	p.E470*	2000.84464
H3F3A	NM_002107.4	c.83A>T	p.K28M	2687.48888
TP53	NM_000546.5	c.991C>T	p.Q331*	Negative
PPM1D	NM_003620.3	c.1430del	p.N477Ifs*6	65
H3F3A	NM_002107.4	c.83A>T	p.K28M	60
DAXX	NM_001141970.1	c.2183G>A	p.R728Q	174
TP53	NM_000546.5	c.317del	p.S106Tfs*17	3370
TERT	NM_198253.2	c.-146C>T	.	1256
H3F3A	NM_002107.4	c.83A>T	p.K28M	1403
RGPD3	NM_001144013.1	c.5114T>A	p.V1705E	Negative
TP53	NM_000546.5	c.524G>A	p.R175H	Negative
TP53	NM_000546.5	c.637C>T	p.R213*	254
TP53	NM_000546.5	c.659A>G	p.Y220C	255
CBL	NM_005188.3	c.1139T>C	p.L380P	280
TP53	NM_000546.5	c.643A>G	p.S215G	18.57206
MAP2K1	NM_002755.3	c.1062A>C	p.Q354H	Negative
				Negative
ACVR1	NM_001105.4	c.617G>A	p.R206H	Negative
KRAS	NM_033360.3	c.35G>A	p.G12D	Negative
RAF1	NM_002880.3	c.1655A>G	p.N552S	Negative
SMO	NM_005631.4	c.536C>T	p.T179M	Negative
TERT	NM_198253.2	c.-124C>T	.	Negative

PIK3CA	NM_006218.3	c.1634A>G	p.E545G	Negative
TERT	NM_198253.2	c.-124C>T	.	Negative
FAT1	NM_005245.3	c.2417G>A	p.R806H	Negative
NF1	NM_000267.3	c.3611G>A	p.R1204Q	Negative
NF1	NM_000267.3	c.6792C>G	p.Y2264*	Negative
TERT	NM_198253.2	c.-124C>T	.	Negative
HMCN1	NM_031935.2	c.9522T>A	p.D3174E	51.3701
YAP1	NM_000548.4	c.991C>T	p.R331W	12.9502
MET	NM_130799.2	c.3965C>A	p.P1322H	36.5211
TERT	NM_198253.2	c.-146C>T	.	Negative
SMO	NM_005631.4	c.536C>T	p.T179M	60.76393
RICTOR	NM_001285439.1	c.2729G>A	p.R910H	100.39684
RGPD3	NM_020975.4	c.2244G>T	p.K748N	36.78885
BRCA1	NM_000489.4	c.3448C>T	p.P1150S	85.17884
				Negative
TSC2	NM_000548.4	c.4286C>T	p.A1429V	Negative
AKT1	NM_001014432.1	c.49G>A	p.E17K	Negative
TERT	NM_198253.2	c.-124C>T	.	Negative
CHEK2	NM_007194.3	c.1116_1117inv	p.K373E	Negative
SMARCA4	NM_001128849.1	c.968C>T	p.S323L	Negative
RAF1	NM_002880.3	c.424-5_424-3del	.	Negative
NF2	NM_000268.3	c.552G>A	p.W184*	Negative
ERBB3	NM_001982.3	c.3383G>A	p.G1128E	6.29622
RGPD3	NM_001144013.1	c.2266A>G	p.N756D	21.31857
ERBB2	NM_004448.3	c.1447G>A	p.D483N	Negative
PIK3CB	NM_006219.2	c.1697G>A	p.R566Q	Negative
HMCN1	NM_031935.2	c.4009A>T	p.I1337F	Negative
SETD2	NM_014159.6	c.1205G>A	p.R402Q	Negative
				Negative
ALK	NM_004304.4	c.1184G>A	p.R395H	46.5605
ERBB2	NM_004448.3	c.688C>T	p.P230S	Negative
FLT3	NM_004119.2	c.964A>G	p.R322G	73.20415
				Negative
FAT1	NM_005245.3	c.2129A>G	p.N710S	Negative
ACVR1	NM_001105.4	c.617G>A	p.R206H	Negative
CBL	NM_005188.3	c.1259G>A	p.R420Q	11.75337
ROS1	NM_002944.2	c.6639C>A	p.D2213E	36.77292
TP53	NM_000546.5	c.643A>G	p.S215G	14.15456
BRCA1	NM_007294.3	c.397C>T	p.R133C	51.3352
CBL	NM_005188.3	c.2569C>T	p.L857F	10.3952
				Negative
				Negative
PIK3R1	NM_006219.2	c.1353_1355delATA	p.E451_Y452delinsD	Negative
FGFR3	NM_005245.3	c.713G>A	p.R238Q	Negative
FGFR1	NM_005245.3	c.304G>A	p.V102I	88.38656
				Negative
				Negative
				Negative
				Negative
				Negative
				Negative

VAF in CSF	Depth in tissue	VAF in tissue	Cosmic
Negative	1385.24619	0.3893	COSV64731746;OCCURENCE=398
0.3592	1774.8033	0.3914	COSV64731746;OCCURENCE=398
0.3924	1858.69155	0.4099	COSV55430916;OCCURENCE=1
0.4306	3811.7007	0.8406	COSV62204363;OCCURENCE=5
0.6561	2252.7396	0.4968	COSV59956007;OCCURENCE=2
0.2994	3035.20048	0.8528	COSV64731746;OCCURENCE=398
Negative	1769.22861	0.4971	COSV50636760;OCCURENCE=2
Negative	1672.42109	0.4699	COSV54941911;OCCURENCE=1
Negative	2200.59153	0.6183	COSV67627551;OCCURENCE=1
0.3249	1520.44752	0.4272	COSV52660980;OCCURENCE=1188
0.1705	202.13454	0.0281	COSV99081608;OCCURENCE=1
0.3621	2751.4755	0.3825	COSV54046205;OCCURENCE=1
0.3778	3612.52548	0.5022	COSV64731746;OCCURENCE=398
Negative	6794.88564	0.9446	COSV52662066;OCCURENCE=1133
0.1053	2674.4383	0.4505	COSV64871414;OCCURENCE=3
0.349	5451.57978	0.9183	COSV64731746;OCCURENCE=398
0.4053	5601.77576	0.9436	COSV53648704;OCCURENCE=1
0.4925	2856.09826	0.4811	COSV52944494;OCCURENCE=1
Negative	1569.94062	0.3786	COSV64731746;OCCURENCE=426
Negative	1612.65163	0.3889	COSV59956020;OCCURENCE=6
Negative	1743.68735	0.4205	COSV62191410;OCCURENCE=53
0.1033	992.55936	0.2496	COSV64731746;OCCURENCE=426
0.1181	1093.565	0.275	0
0.2479	156.28038	0.0393	COSV52661282;OCCURENCE=596
0.4935	1935.41688	0.6404	COSV64731746;OCCURENCE=426
Negative	2781.9351	0.9205	COSV52661580;OCCURENCE=1344
Negative	866.47092	0.1212	COSV51765841;OCCURENCE=73
0.4885	3342.20425	0.4675	COSV64731746;OCCURENCE=398
0.9714	6575.02727	0.9197	COSV52662066;OCCURENCE=1133
Negative	3437.28728	0.4808	0
0.0795	4463.95258	0.6983	COSV55115924;OCCURENCE=19
0.1096	3024.97832	0.4732	COSV64731746;OCCURENCE=398
Negative	1396.7831	0.2185	COSV55882697;OCCURENCE=14
Negative	4615.18872	0.4908	COSV99068602;OCCURENCE=1
0.1966	4189.2147	0.4455	COSV58682976;OCCURENCE=49
0.2576	5350.5346	0.569	COSV64731746;OCCURENCE=398
Negative	8488.44918	0.9027	COSV52662066;OCCURENCE=1133
0.3928	1693.93848	0.3809	COSV62213436;OCCURENCE=3
0.5276	1656.13728	0.3724	COSV64731746;OCCURENCE=426
Negative	1415.54376	0.3183	COSV52664188;OCCURENCE=112
0.0742	1943	0.2727	COSV59959080;OCCURENCE=1
0.1022	1787	0.2337	COSV64731746;OCCURENCE=426
0.2062	3725	0.5322	.
0.5597	129	0.0305	COSV52752463;OCCURENCE=3
0.3015	428	0.3934	.
0.3394	2652	0.4089	COSV64731746;OCCURENCE=426
Negative	168	0.0536	COSV58744427;OCCURENCE=1
Negative	1472	0.5534	COSV52661038;OCCURENCE=1879
0.023	2105	0.3299	COSV52665560;OCCURENCE=728
0.0242	1969	0.3263	COSV52661282;OCCURENCE=596
0.0571	118	0.0172	COSV50630049;OCCURENCE=24
0.1171	Negative	Negative	COSV52675946;OCCURENCE=37
Negative	3079.56168	0.4734	0
Negative	Negative	Negative	
Negative	1619.17056	0.1746	COSV55115924;OCCURENCE=19
Negative	3051.0144	0.329	COSV55497369;OCCURENCE=15631
Negative	4522.73472	0.4877	COSV50105458;OCCURENCE=1
Negative	5708.82816	0.6156	COSV50853584;OCCURENCE=1
Negative	3079.76256	0.3321	0

Negative	3416.41589	0.3203	COSV55873220;OCCURENCE=185
Negative	3487.8801	0.327	0
Negative	2137.5159	0.481	COSV71675921;OCCURENCE=2
Negative	1226.96079	0.2761	0
Negative	1189.18764	0.2676	COSV62214006;OCCURENCE=2
Negative	1584.69474	0.3566	0
0.7037	Negative	Negative	COSV99628350;OCCURENCE=1
0.1774	Negative	Negative	COSV56772552;OCCURENCE=3
0.153	Negative	Negative	COSV59260203;OCCURENCE=1
Negative	1995.92644	0.3572	0
0.3721	Negative	Negative	COSV50853584;OCCURENCE=1
0.6148	Negative	Negative	COSV57125248;OCCURENCE=4
0.0945	Negative	Negative	COSV104393606;OCCURENCE=2
0.2188	Negative	Negative	COSV100525526;OCCURENCE=1
Negative	Negative	Negative	
Negative	4308.34108	0.5009	COSV51925502;OCCURENCE=1
Negative	1058.80772	0.1231	COSV62571334;OCCURENCE=748
Negative	1683.25484	0.1957	0
Negative	311.96567	0.1721	COSV60415399;OCCURENCE=27
Negative	381.57335	0.2105	0
Negative	744.83843	0.4109	0
Negative	1563.09121	0.8623	COSV58522627;OCCURENCE=2
0.0526	Negative	Negative	COSV57265491;OCCURENCE=1
0.1781	Negative	Negative	COSV58738024;OCCURENCE=15
Negative	3222.1332	0.6435	COSV54087680;OCCURENCE=1
Negative	3267.198	0.6525	0
Negative	3161.04536	0.6313	COSV54902576;OCCURENCE=1
Negative	3130.70043	0.3289	COSV57431930;OCCURENCE=3
Negative	Negative	Negative	
0.371	4425.25048	0.4769	COSV66557504;OCCURENCE=12
Negative	4442.88096	0.4788	COSV99068602;OCCURENCE=1
0.5833	8515.52184	0.9177	0
Negative	Negative	Negative	
Negative	1896.01202	0.5059	COSV71674607;OCCURENCE=2
Negative	797.798	0.295	COSV55115924;OCCURENCE=19
0.0909	Negative	Negative	COSV50629675;OCCURENCE=27
0.2844	Negative	Negative	COSV63856735;OCCURENCE=2
0.0994	Negative	Negative	COSV52675946;OCCURENCE=37
0.3605	1882.23954	0.4638	COSV58783472;OCCURENCE=2
0.073	Negative	Negative	COSV50654489;OCCURENCE=1
Negative	Negative	Negative	
Negative	Negative	Negative	
Negative	1801.14592	0.3451	0
Negative	2446.23904	0.4687	0
0.3904	Negative	Negative	COSV58330448;OCCURENCE=2
Negative	Negative	Negative	
Negative	Negative	Negative	
Negative	Negative	Negative	
Negative	Negative	Negative	
Negative	Negative	Negative	
Negative	Negative	Negative	

Annotation	IMPACT	Manual filterate
missense_variant	MODERATE	1
missense_variant	MODERATE	1
missense_variant	MODERATE	1
stop_gained	HIGH	1
stop_gained	HIGH	0
missense_variant	MODERATE	1
missense_variant	MODERATE	1
missense_variant	MODERATE	0
missense_variant	MODERATE	0
missense_variant	MODERATE	1
missense_variant	MODERATE	0
missense_variant	MODERATE	0
missense_variant	MODERATE	1
missense_variant	MODERATE	1
stop_gained	HIGH	1
missense_variant	MODERATE	1
missense_variant	MODERATE	0
splice_region_variant&intron_variant	MODIFIER	1
missense_variant	MODERATE	1
stop_gained	MODERATE	1
frameshift_variant	HIGH	1
missense_variant	MODERATE	1
frameshift_variant	HIGH	1
missense_variant	MODERATE	1
missense_variant	MODERATE	1
missense_variant	MODERATE	1
missense_variant	MODERATE	1
missense_variant	MODERATE	1
missense_variant	MODERATE	1
missense_variant&splice_region_variant	MODERATE	1
missense_variant	MODERATE	1
missense_variant	MODERATE	1
missense_variant	MODERATE	1
missense_variant	MODERATE	1
stop_gained	HIGH	1
missense_variant	MODERATE	1
missense_variant	MODERATE	1
stop_gained	HIGH	1
missense_variant	MODERATE	1
stop_gained&splice_region_variant	HIGH	1
frameshift_variant	HIGH	0
missense_variant	MODERATE	1
missense_variant	MODERATE	1
frameshift_variant	HIGH	1
upstream_gene_variant	MODIFIER	1
missense_variant	MODERATE	1
missense_variant	MODERATE	0
missense_variant	MODERATE	1
stop_gained	HIGH	1
missense_variant	MODERATE	1
missense_variant	MODERATE	1
missense_variant	MODERATE	1
missense_variant	MODERATE	1
missense_variant	MODERATE	1
missense_variant	MODERATE	1
missense_variant	MODERATE	0
missense_variant&splice_region_variant	MODERATE	1
upstream_gene_variant	MODIFIER	1

missense_variant	MODERATE	1
upstream_gene_variant	MODIFIER	1
missense_variant	MODERATE	1
missense_variant	HIGH	1
stop_gained	HIGH	0
upstream_gene_variant	MODIFIER	1
missense_variant	MODERATE	0
missense_variant	MODERATE	1
missense_variant	MODERATE	0
upstream_gene_variant	MODIFIER	1
missense_variant&splice_region_variant	MODERATE	1
missense_variant	MODERATE	1
missense_variant	MODERATE	0
missense_variant	HIGH	1
missense_variant	MODERATE	0
missense_variant&splice_region_variant	MODERATE	1
upstream_gene_variant	MODIFIER	1
missense_variant	MODERATE	0
missense_variant	MODERATE	1
splice_region_variant&intron_variant	MODERATE	1
stop_gained	HIGH	1
missense_variant	MODERATE	1
missense_variant	MODERATE	0
missense_variant	MODERATE	1
missense_variant	MODERATE	1
missense_variant	MODERATE	0
missense_variant	MODERATE	1
missense_variant	MODERATE	1
missense_variant	MODERATE	1
missense_variant	MODERATE	1
missense_variant	MODERATE	1
missense_variant	MODERATE	1
missense_variant	MODERATE	1
missense_variant	MODERATE	1
missense_variant	MODERATE	1
missense_variant	MODERATE	0
missense_variant	MODERATE	0
disruptive_inframe_deletion	MODERATE	1
missense_variant	MODERATE	1
missense_variant	MODERATE	1

Supplementary Table 4. CNVs identified in tumor tissue and cerebrospinal fluid.

Sample	Shareded situation	Gene	CN in CSF	CN in tissue	Type
3	Shared	CDK4	5.37	6.04	Amplification
3	Shared	MDM2	4.32	5.52	Amplification
4	Shared	KDR	3.52	3.53	Amplification
4	Tissue only	CDK6	Negative	2.95	Amplification
4	Tissue only	PTEN	Negative	1.36	Deletion
5	Tissue only	KDR	Negative	2.74	Amplification
5	Tissue only	MET	Negative	4.15	Amplification
5	Tissue only	TSC2	Negative	0.68	Deletion
5	Tissue only	TRAF7	Negative	0.7	Deletion
6	Tissue only	PIK3CA	Negative	3.24	Amplification
9	Shared	PDGFRA/KIT	20.06/21.99	21.79/22.07	Amplification
9	Shared	VEGFR2	8.01	9.74	Amplification
9	Shared	TERT	4.93	5.57	Amplification
9	Shared	KLF4	4.46	3.58	Amplification
9	Shared	NTRK3	3.56	4.77	Amplification
9	Tissue only	PIK3R1	Negative	4.17	Amplification
9	Tissue only	FLT4	Negative	0.97	Deletion
9	Tissue only	CDK6	Negative	3.29	Amplification
9	Tissue only	CDKN2A	Negative	1.19	Deletion
9	Tissue only	IDH2	Negative	3.57	Amplification
11	CSF only	CDK6	3.48	Negative	Amplification
11	Shared	MDM2	4.27	3.89	Amplification
11	Shared	DICER1	0.75	0.8	Deletion
11	Shared	ERBB2	3.92	4.05	Amplification
12	Tissue only	RGPD3	Negative	4.26	Amplification
12	Tissue only	BCL2L11	Negative	4.2	Amplification
12	Tissue only	MYC	Negative	3.59	Amplification
12	Tissue only	CDK4	Negative	3.19	Amplification
13	Shared	PDGFRA/KIT	3.97	4.24	Amplification
13	Shared	KRAS	23.53	17.16	Amplification
16	CSF only	NF1	0.68	Negative	Deletion
16	Tissue only	MET	Negative	4.8	Amplification
17	Tissue only	VEGFR2	Negative	3.3	Amplification
17	Tissue only	CDK4	Negative	14	Amplification
18	Tissue only	IRS2	Negative	0.93	Deletion
20	Tissue only	CDK6	Negative	3.05	Amplification
22	Tissue only	ALK	Negative	3.73	Amplification
22	Tissue only	PLCG1	Negative	3.39	Amplification
26	Tissue only	MDM4	Negative	3.01	Amplification
30	Tissue only	RGPD3	Negative	4.86	Amplification
31	Tissue only	RGPD3	Negative	4.02	Amplification
32	Tissue only	CDK6	Negative	3.39	Amplification
32	Tissue only	CDK4	Negative	3.41	Amplification
32	Tissue only	MDM2	Negative	3.29	Amplification
34	Tissue only	RGPD3	Negative	3.05	Amplification
34	Tissue only	CDK6	Negative	3	Amplification
34	Tissue only	IRS2	Negative	0.98	Deletion
35	Tissue only	CHEK2	Negative	0.93	Deletion
35	Tissue only	IRS2	Negative	0.88	Deletion
35	Tissue only	CDK6	Negative	3.56	Amplification
37	Tissue only	RGPD3	Negative	4.61	Amplification
